# Altered brain fluid management in a rat model of arterial hypertension

**DOI:** 10.1186/s12987-020-00203-6

**Published:** 2020-06-26

**Authors:** Daphne M. P. Naessens, Bram F. Coolen, Judith de Vos, Ed VanBavel, Gustav J. Strijkers, Erik N. T. P. Bakker

**Affiliations:** Department of Biomedical Engineering and Physics, Amsterdam Cardiovascular Sciences, Amsterdam Neuroscience, University of Amsterdam, Meibergdreef 9, 1105 AZ Amsterdam, The Netherlands

**Keywords:** Aquaporin 4, Cerebral oedema, Cerebrospinal fluid, Hypertension, Interstitial fluid

## Abstract

**Background:**

Proper neuronal function is directly dependent on the composition, turnover, and amount of interstitial fluid that bathes the cells. Most of the interstitial fluid is likely to be derived from ion and water transport across the brain capillary endothelium, a process that may be altered in hypertension due to vascular pathologies as endothelial dysfunction and arterial remodelling. In the current study, we investigated the effects of hypertension on the brain for differences in the water homeostasis.

**Methods:**

Magnetic resonance imaging (MRI) was performed on a 7T small animal MRI system on male spontaneously hypertensive rats (SHR) and normotensive Wistar Kyoto rats (WKY) of 10 months of age. The MRI protocol consisted of T2-weighted scans followed by quantitative apparent diffusion coefficient (ADC) mapping to measure volumes of different anatomical structures and water diffusion respectively. After MRI, we assessed the spatial distribution of aquaporin 4 expression around blood vessels.

**Results:**

MRI analysis revealed a significant reduction in overall brain volume and remarkably higher cerebroventricular volume in SHR compared to WKY. Whole brain ADC, as well as ADC values of a number of specific anatomical structures, were significantly lower in hypertensive animals. Additionally, SHR exhibited higher brain parenchymal water content. Immunohistochemical analysis showed a profound expression of aquaporin 4 around blood vessels in both groups, with a significantly larger area of influence around arterioles. Evaluation of specific brain regions revealed a decrease in aquaporin 4 expression around capillaries in the corpus callosum of SHR.

**Conclusion:**

These results indicate a shift in the brain water homeostasis of adult hypertensive rats.

## Background

A well-regulated water homeostasis in the brain is of vital importance for proper neuronal function. Brain parenchymal cells bathe in the interstitial fluid (ISF), which acts as a medium for both nutrient delivery and waste removal, while also providing the ionic environment for neuronal activity. The source of ISF, the presence of flow in the interstitial space, as well as its interaction with cerebrospinal fluid (CSF) via paravascular channels, are an area of debate [1–3]. However, the abundant expression of the water channel aquaporin 4 (AQP4) around both blood vessels and interfaces between ISF and CSF, suggest extensive exchange of water between those fluid compartments. Disturbances in the composition, turnover and amount of ISF are profound in acute conditions such as ischemic stroke [4]. Also more chronic neuropathologies as Alzheimer’s disease (AD) and vascular dementia may be associated with changes in ISF turnover, as suggested by impaired clearance of waste substances like amyloid-β (Aβ) from the brain interstitium [5].

Chronic hypertension has been identified as an important risk factor for neurodegenerative diseases [6]. Particularly mid-life hypertension is associated with cognitive decline later in life. However, the aetiology of hypertensive brain damage is not clear. Both structural and functional changes in the cerebral vasculature such as arterial remodelling, loss of blood–brain barrier (BBB) integrity, and endothelial dysfunction have been described [6]. For example, the media to lumen ratio was found to be increased in cerebral resistance arteries of hypertensive patients [7]. Another study showed that cerebral blood flow is reduced in several regions of the brain in elderly hypertensives [8]. Thus, while a number of cerebrovascular pathologies have been associated with high blood pressure, the mechanisms by which hypertension contributes to brain damage are still not fully understood.

The spontaneously hypertensive rat (SHR) shows representative symptoms of human chronic hypertension, including cognitive impairment, endothelial dysfunction and vascular remodelling [9, 10]. Furthermore, this strain exhibits a number of neurodegenerative changes [11], increased deposition of Aβ, impaired ISF-CSF exchange, and CSF reflux into the ventricular system [12, 13]. Even though these studies suggest a disturbed ISF and/or CSF homeostasis, recent work by our group showed unaltered CSF production, intracranial pressure, and integrity of the BBB with respect to small molecules in this hypertensive rat model [14]. Therefore, in the current study, we studied the impact of hypertension on the brain for other changes in water homeostasis. Magnetic resonance imaging (MRI) was performed to acquire anatomical scans in vivo and measure ventricular and brain tissue volumes. In addition, diffusion weighted imaging (DWI) scans were made to detect cerebral oedema. As a second approach to assess brain oedema, we determined the brain parenchymal water content. Since water homeostasis depends on AQP4, we lastly quantified the total AQP4 expression and its spatial distribution around blood vessels.

## Methods

### Animals

A total of 11 male normotensive Wistar Kyoto rats (WKY/NHsd) and 11 male spontaneously hypertensive rats (SHR/NHsd) were enrolled in this study. One SHR died before the experimental procedure, leaving 11 rats in the normotensive control group and 10 rats in the hypertensive group. All animals were obtained from Envigo (United Kingdom) and were 6 weeks of age upon arrival in the animal facility. They were kept until 45 ± 0.2 weeks of age for the experimental procedure. Rats were housed in groups in individually ventilated cages on a 12-h light/12-h dark schedule and had ad libitum access to food and water. All experiments were conducted in accordance with the ARRIVE guidelines and European Union guidelines for the welfare of laboratory animals (Directive 2010/63/EU), and were approved by the Academic Medical Center Animal Ethics Committee.

### Blood pressure and heart rate measurements

One week prior to the MR experiments, blood pressure and heart rate were measured in conscious rats using a non-invasive tail-cuff system (Kent Scientific). Rats were accustomed to the procedure by placing them in the restrainer for 10 min on 4 consecutive days. In order to measure the blood pressure non-invasively, animals were placed in the restrainer on a warming pad. The occlusion cuff and volume pressure recording sensor were subsequently placed on the tail. Once the blood volume in the tail was sufficient, 10 blood pressure and heart rate measurements were recorded from each individual rat and averaged.

### MR imaging

MR imaging was performed on a 7T small animal MRI system (MR Solutions, Guildford, United Kingdom) using a dedicated rat head coil (MR Solutions). Animals were weighed prior to the MRI experiments. Then, animals were anaesthetized with 2.5–3.0% isoflurane (Pharmachemie B.V.) in a mixture of 0.5 L/min medical air and 0.5 L/min O_2_ using an induction chamber. Ophthalmic ointment (Systane Nighttime, Alcon) was applied to prevent dehydration of the eyes during the MRI session. Rats were subsequently placed on the MRI cradle in prone position. The head was immobilized using a bite bar and ear bars to minimize movement of the head during the image acquisition. Maintenance isoflurane inhalation anaesthesia was applied via the nose mask of the cradle and was kept between 2.0 and 2.5% in a mixture of 0.4 L/min medical air and 0.4 L/min O_2_. The isoflurane concentration was manually adjusted during the procedure to maintain a respiration rate of 45–55 breaths per minute. This was monitored using a pressure balloon placed at the animal’s abdomen. Additionally, the core body temperature was measured with a rectal probe and was maintained at 36–37 °C via the animal bed.

Scout images of the brain were acquired for localisation of the following scans. Subsequently, anatomical multi-slice T2-weighted (T2w) turbo-spin echo scans were performed in coronal direction using the following sequence parameters: TR/TE = 4000/45 ms, flip angle = 90°, echo-train length ETL = 7, FOV = 35 × 35 mm^2^, matrix size = 256 × 256, slice thickness = 1 mm, number of slices = 26, NSA = 4, total acquisition time = 9 min. For quantitative apparent diffusion coefficient (ADC) mapping, multi-slice diffusion weighted imaging (DWI) was performed in 3 orthogonal gradient directions using a single echo spin-echo sequence with the following parameters: TR/TE = 2000/30 ms, flip angle = 90°, FOV = 35 × 35 mm^2^, b-values = 0/800 s/mm^2^, matrix size = 128 × 128, slice thickness = 1 mm, number of slices = 5, NSA = 1, total acquisition time = 13 min. At the end of the MRI scanning protocol, animals were sacrificed with an overdose of Euthasol^®^ via the tail vein catheter.

### Brain water content

After sacrifice, the brains were carefully removed from the skull and the whole brain wet weight was determined. Then, the brains were divided into its two hemispheres. The left hemisphere was snap frozen in liquid nitrogen and stored at −80 °C until further use. The right hemisphere was used to determine the water content in the brain parenchyma. To this end, the cerebrospinal fluid was removed from the lateral and third ventricles, as well as from the surface on the cerebrum and cerebellum, using absorbent swabs. The hemisphere weights were subsequently determined before and after desiccation at 90 °C for 7 days from which the brain water content percentage could be calculated as:

$$\% \,Water\,content\, = \,\frac{{\left( {Wet\,brain\,weight\, - \,Dry\,brain\,weight} \right)}}{Wet\,brain\,weight}\, \times \,100$$

As described in the study by Keep et al. [15], a relatively small change in the percentage brain water content can reflect a much larger change in the actual brain oedema formation. Therefore, the water content of the brain was also calculated using the following equation:

$$Water\,content = \frac{{\left( {Wet\,brain\,weight - Dry\,brain\,weight} \right)}}{Dry\,brain\,weight}$$

### MRI data analysis

**Fig. 1:**
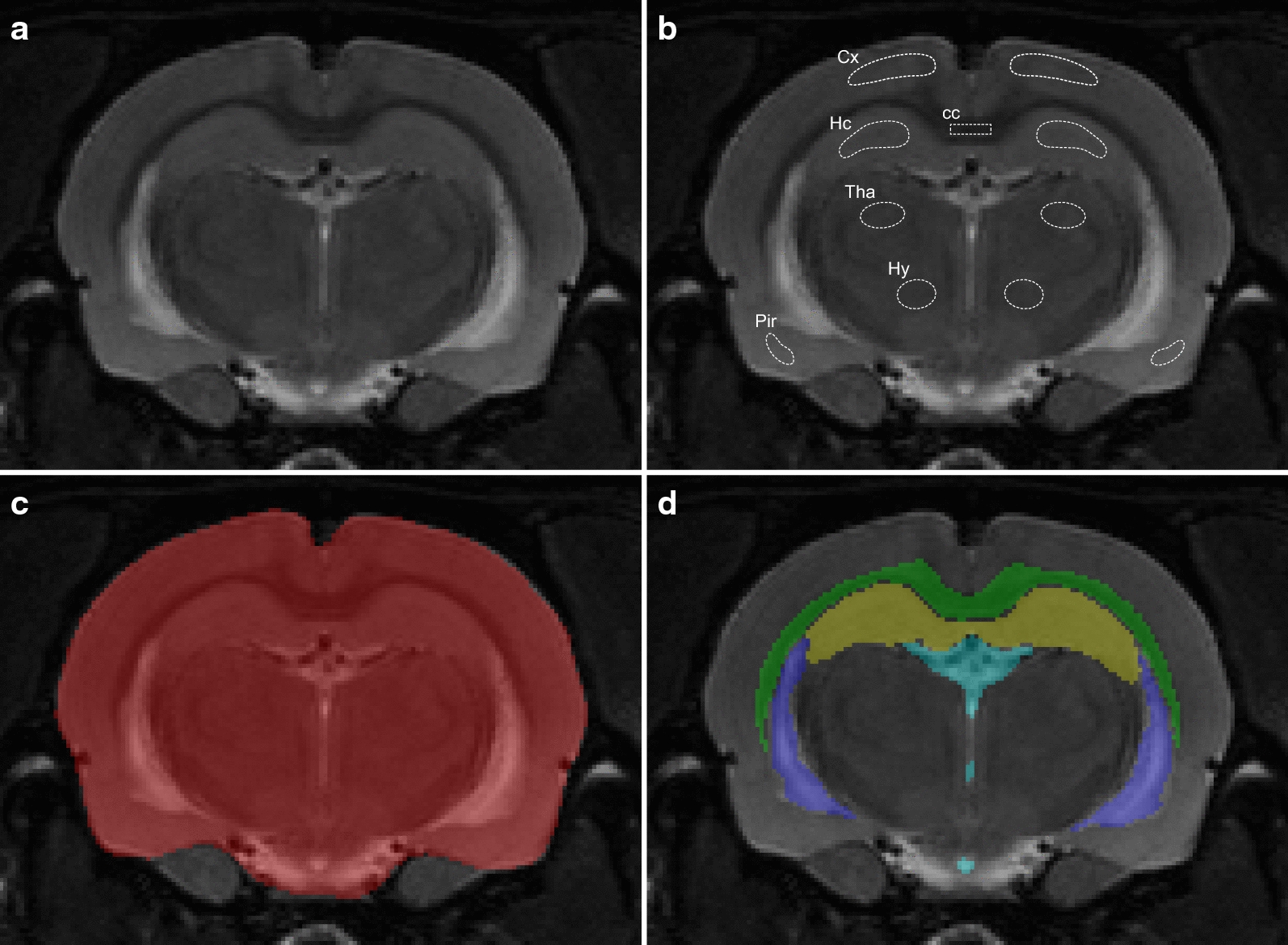
Manual segmentation of different anatomical structures. **a** Representative slice from the multi-slice T2w scan of a WKY rat. **b** Region of interest (ROI) selection in different anatomical brain structures used for ADC quantification. cc, corpus callosum; Cx, cerebral cortex; Hc, hippocampus; Tha, thalamus; Hy, hypothalamus; Pir, piriform cortex. **c** Segmentation of total intracranial volume. **d** Segmentation of different anatomical brain structures used for volume quantification. Green, corpus callosum; Yellow, hippocampus; Light blue, Third ventricle; Dark blue, lateral ventricle

Whole brain as well as specific anatomical structures (corpus callosum, hippocampus, lateral ventricle and third ventricle) were manually segmented by one single observer (D.N.) on the multi-slice coronal T2w images using ITK-SNAP (version 3.6.0). For each segmented region, the total number of pixels from all slices was multiplied by the voxel volume to quantify brain region volumes. Representative images of the manual segmentation are shown in Fig. 1c, d. For ADC quantification, DWI images were first registered and interpolated to the T2w images using FSL 6.0 [16]. Regions of interest (ROIs) within the brain parenchyma were manually drawn by one single observer (D.N.) using ITK-SNAP in a single slice of the T2w corresponding to the centre slice from the diffusion weighted scans (Fig. 1a, b). Care was taken not to include voxels of adjacent brain structures, as this could affect the ADC value for that specific brain region. In each ROI, ADC values were calculated by fitting a mono-exponential decay function to the DW signals acquired at different b-values and averaging over the three diffusion gradient directions.

### Immunohistochemistry and quantification

The left hemisphere was divided into 3 coronal blocks using an adult rat brain slicer (Zivic Instruments). The middle part, containing the hippocampus, was subsequently embedded in TissueTek^®^ and snap frozen in liquid nitrogen. Coronal slices of 5 µm thickness were cut on a cryostat (Microm HM 560) and collected on SuperFrost glass slides and stored at −80 °C until further use. For fluorescent double immunostaining, slides were allowed to acclimatize to room temperature for 30 min. Brain sections were fixed with 4% paraformaldehyde for 30 min and subsequently rinsed in phosphate buffered saline (PBS, pH 7.35). Slides were blocked for 1 h at room temperature with 3% bovine serum albumin (BSA, Sigma) containing normal goat serum (5%) and Triton X-100 (0.05%). This was followed by simultaneous incubation with the primary antibodies against rabbit anti-aquaporin 4 (AQP4, 1:500, Millipore) and mouse anti-smooth muscle myosin heavy chain II (Myosin, 1:500, Abcam) for AQP4 and myosin double immunostaining, and mouse anti-glial fibrillary acidic protein (GFAP, 1:200, Bio-Rad) for GFAP immunostaining, overnight at 4 °C. The next day, sections were rinsed in PBS and incubated with Cy3-conjugated goat anti-rabbit (1:300, Brunschwig) and Cy5-conjugated goat anti-mouse (1:300, Invitrogen) secondary antibodies for 1 h at room temperature for AQP4 and myosin staining. For GFAP immunostaining, sections were rinsed in PBS and incubated with a Cy3-conjugated goat anti-mouse (1:300, Brunschwig) secondary antibody for 2 h at room temperature. To visualize the cell nuclei, slides were washed with PBS and incubated in bisbenzimide (1:100, 3.5 mg/ml, Sigma) for 3 min. After a final wash, fluorescent mounting medium (DAKO) was used to coverslip the sections. Detailed and overview images of the hemisphere were acquired with a confocal laser scanning microscope (Leica TCS SP8 SMD and Leica TCS SP8 DLS) with 40× or 20× and 20× or 10× objectives respectively.

The expression of AQP4 along parenchymal blood vessels was quantified from confocal images using ImageJ. Per animal, 5 arterioles, 5 capillaries, and 5 venules were selected in the cortex at random to study differences in the AQP4 expression among different types of blood vessels and animal type. This selection was performed by a researcher who was blinded to the animal strain. Blood vessels were marked as arterioles when the diameter along the shortest axis was larger than 20 µm and if myosin staining was observed. In case myosin immunoreactivity was missing in the same sized vessels, these vessels were marked as venules. To study differences in the AQP4 expression between normotensive and hypertensive rats in different brain structures (Fig. 1b), 5 capillaries were randomly selected in each brain region. A straight line was drawn of 50 µm long and 8.5 µm wide, perpendicular to the longest axis of the blood vessel. In order to include the AQP4 signal from the vascular astrocyte endfeet and surrounding parenchyma, the starting point of the line was placed at the first AQP4 immunoreactive pixel as seen from the lumen. The intensity profile of each blood vessel was plotted, normalized to the AQP4 background intensity, background corrected and subsequently averaged per animal. In addition, the area under the curve (AUC) of these mean intensity profiles was calculated. To assess reactive changes in the astroglial compartment, GFAP immunoreactivity was quantified in all different brain regions using ImageJ. Anatomical brain structures were manually annotated and the mean pixel intensity was measured to determine the immunofluorescence signal.

### Lysate preparation and analysis of AQP4 expression with Wes

To quantitatively measure the AQP4 expression in the brain parenchyma, the ProteinSimple Wes system was used. Protein lysates from brain tissue were obtained by dissection of a small fragment of approximately 75 mg from the frontal tissue block of the left hemisphere. Therefore, samples mainly consisted of the cerebral cortex. Brain tissues were mechanically homogenized in radioimmunoprecipitation assay buffer (RIPA, 150 mM NaCl, 1.0% NP40, 0.5% sodium deoxycholate, 0.1% SDS, 50 mM Tris, 1 mM EDTA, pH 8.0) containing a protease inhibitor cocktail (Roche) using a Potter–Elvehjem tissue grinder. To obtain a fully homogenous solution, lysates were subsequently granulated with an automated homogenizer (Kinematica) and were left on ice for an additional 30 min. Then, samples were centrifuged at 5000 rpm for 30 min and the supernatant was carefully extracted.

To determine the total protein concentration, lysates were diluted 100× with MilliQ water to make them compatible with the Qubit™ protein assay kit (Molecular Probes, Life Technologies). Concentrations were measured according to the manufacturer’s instructions. An equally diluted RIPA buffer sample was used to measure the background signal and was subsequently subtracted from all protein concentrations of the tissue homogenates. Samples were further diluted with MilliQ to a final concentration of 1 mg/ml and were sonicated for 10 s at 30 Hz. Brain lysates were stored at −80 °C upon further use.

For the Wes application, samples were prepared according to the manufacturer’s instructions of the 12−230 kDa Wes Separation Module (ProteinSimple SM-W004). Pre-diluted brain lysates were further diluted with 5X Fluorescent Master Mix to a final loading concentration of 0.8 mg/ml. Biotinylated ladder and samples were vortexed and heated at 95 °C for 5 min. Subsequently, lysates and the biotinylated ladder were pipetted into the immunoassay plate as well as the blocking reagent, primary and secondary antibodies, chemiluminescent substrate and wash buffer according to the scheme provided with the separation module. To determine the AQP4 expression in the brain lysates, a rabbit anti-AQP4 (1:100, AB2218, Millipore) primary antibody was used. In addition, a rabbit anti-beta actin (1:50, AB8227, Abcam) primary antibody was used as a loading control. Primary antibodies were subsequently detected with the anti-rabbit detection module (DM-001, ProteinSimple) that is specifically designed for the Wes system. Default settings of the Wes were used which included a separation time of 25 min at 375 Volts, blocking step of 5 min, incubation with primary and secondary antibodies of 30 min each, and chemiluminescence detection with the high dynamic range (HDR; exposures of 1 – 2 – 4 – 8 – 16 – 32 – 64 – 128 – 512 s). The resulting electropherogram data for both sample proteins and fluorescent standards were inspected and manually corrected in case an incorrect peak was identified as a standard. Subsequently, total areas of the 34 kDa and 48 kDa peaks were quantified, which represent protein concentrations of AQP4 and beta-actin respectively. Representative images of both the electropherogram and corresponding Western blot-like image are shown in Additional file 1: Figure S1. Based on 4 Wes experiments, the average AQP4/beta-actin ratio was calculated for each individual animal.

### Statistical analysis

All data are plotted using Tukey boxplots, where the box contains the values for the 25th and 75th percentile of the data. The median is defined with the black line, and the whiskers extend to the minimum and maximum. Each point in the box plot represents an individual animal. All data values are reported as mean ± SEM. Data sets were first tested for normality by the Shapiro–Wilk test. An unpaired Student’s *t* test was performed to compare the means of normally distributed data. The Mann–Whitney U test was applied to test for differences between groups that were not normally distributed. Since the AQP4 expression around capillaries in six different brain regions showed a clear outlier in the data, a Grubbs’ test with a significance level of 0.05 was performed. This resulted in the exclusion of a total of 4 values in the whole dataset (a total of 125 values). AQP4 expression around three different types of blood vessels and different brain regions, GFAP intensity as well as ADC values in six brain regions were analysed using two-way ANOVA, followed by Bonferroni’s post hoc tests. Differences between groups were considered significant at p < 0.05. All statistical analyses were done using GraphPad Prism Software (version 8.0.2).

## Results

### Physiological parameters and brain water content

Prior to the experimental procedure, blood pressure and heart rate were measured non-invasively. Spontaneously hypertensive rats (SHR) had significantly elevated systolic and diastolic blood pressure levels when compared to the normotensive control animals (WKY). Additionally, heart rate was significantly higher in SHR. At 45 ± 0.2 weeks of age, body weight did not differ between the two rat strains. However, the wet brain weight was significantly smaller in SHR as compared to WKY (Table 1).

**Table 1:** Systolic and diastolic blood pressure, heart rate, body and wet brain weights

	WKY (n = 11)	SHR (n = 10)
Blood pressure and heart rate		
Systolic (mmHg)	147.8 ± 5.6	198.5 ± 6.8 ***
Diastolic (mmHg)	98.3 ± 5.2	146.9 ± 9.4 ***
Heart rate (bpm)	419.2 ± 8.9	446.5 ± 7.5 *
Weight		
Body (g)	432.1 ± 9.2	452.9 ± 10.2
Brain (g)	2.21 ± 0.03	2.06 ± 0.02 ***

*WKY* Wistar Kyoto rat, *SHR* spontaneously hypertensive rat

Values are mean ± SEM. *p ≤ 0.05, ***p ≤ 0.001 (unpaired Student’s t-test)

**Fig. 2:**
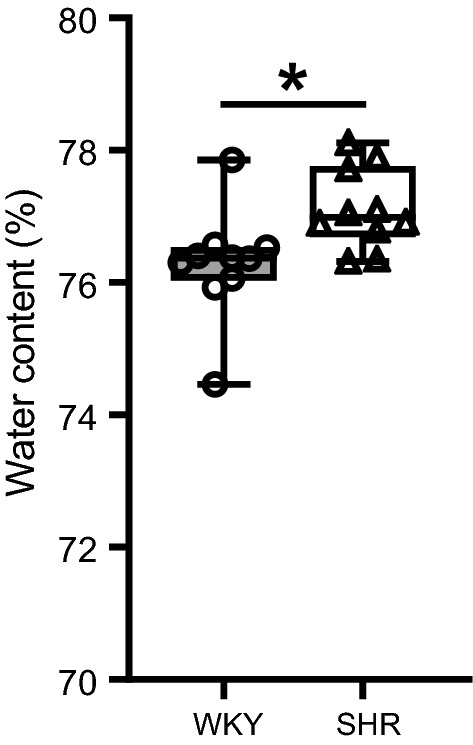
Percentage brain parenchymal water content in WKY and SHR rats. Percentage brain parenchymal water content was significantly elevated in SHR (n = 10) as compared to WKY (n = 10) rats. The boxplots indicate the median and values of the 25th and 75th percentile of the data. * p ≤ 0.05 (Mann–Whitney U test)

As shown in Fig. 2, the % water content in the brain parenchyma was slightly elevated in hypertensive rats with mean values of 77.1 ± 0.2% in SHR vs. 76.3 ± 0.3% in WKY. Even though the difference in % water content between the two strains was small (< 1%), statistical analysis revealed that it was significantly different. Next to this, brain water content was also calculated when normalizing to the dry weight. This revealed water contents of 3.37 ± 0.04 g water/g dry weight in hypertensive rats compared to 3.22 ± 0.05 g water/g dry weight in the normotensive rats, reflecting a 4.7% increase in water content in SHR (p ≤ 0.05).

### Volume measurements of anatomical structures

**Fig. 3:**
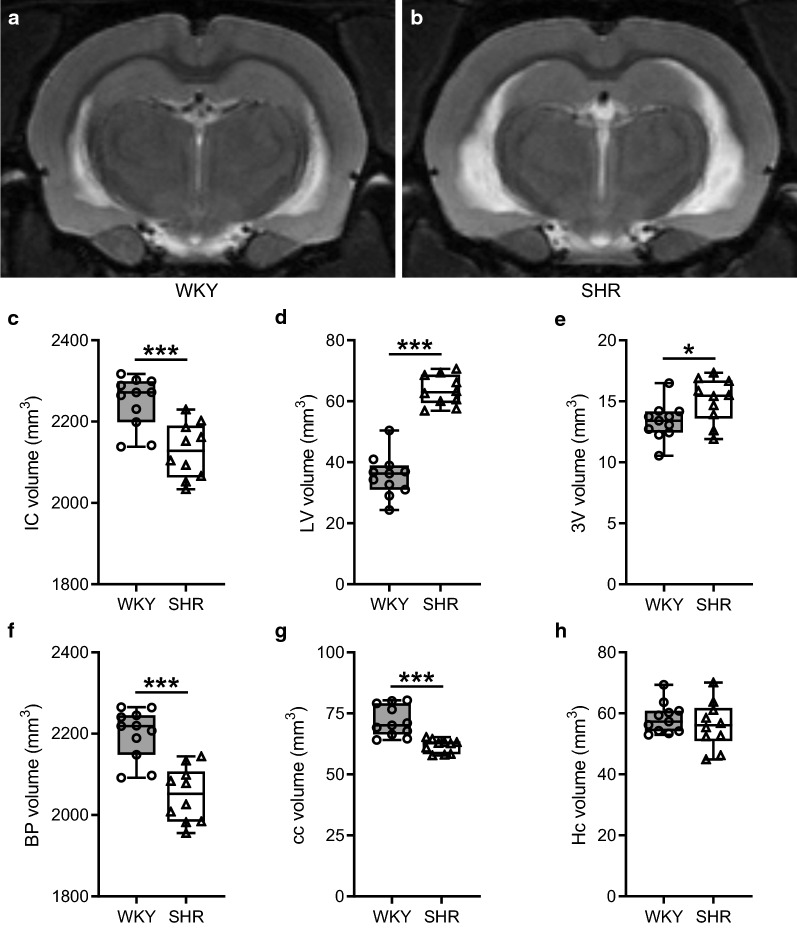
Volume measurements of different anatomical structures. Panels **a**, **b** show a single slice of a multi-slice T2w scan of WKY and SHR rats respectively. **c** Total intracranial (IC) volume was significantly smaller in SHR compared to WKY. **d, e** Lateral ventricle (LV) and third ventricle (3V) volumes were significantly larger in SHR. **f** The brain parenchymal (BP) volume was remarkably less in the hypertensive animals. **g, h** Corpus callosum (cc) was smaller in SHR compared to WKY, while the hippocampus (Hc) volume was not different between the two strains. n = 11 for WKY and n = 10 for SHR. The boxplots indicate the median and values of the 25th and 75th percentile of the data. * p ≤ 0.05, *** p ≤ 0.001 (unpaired Student’s t-test)

T2-weighted anatomical scans were made to study the impact of hypertension on the morphology of different anatomical structures. Figure 3a, b show the same stereotaxic coordinates of a single slice from the multi-slice T2w scans in respectively WKY and SHR rats. Total intracranial brain volumes were significantly smaller in SHR compared to WKY (Fig. 3c). In contrast, both the lateral and third ventricles were larger in the hypertensive animals (Fig. 3d, e). The lateral ventricle volume was nearly twice as large in SHR (63.6 ± 1.6 mm^3^) versus WKY (35.6 ± 2.1 mm^3^). From this, we could calculate the brain parenchymal volume by subtracting the total ventricle volume from the total intracranial volume. As shown in Fig. 3f, SHR exhibited a decrease in total brain tissue volume of about 7%. The volume of the corpus callosum was smaller in SHR (61.7 ± 0.9 mm^3^) compared to WKY (71.8 ± 1.9 mm^3^). Lastly, hippocampal volumes were similar in both strains.

### ADC quantification in the whole brain and different anatomical regions

**Fig. 4:**
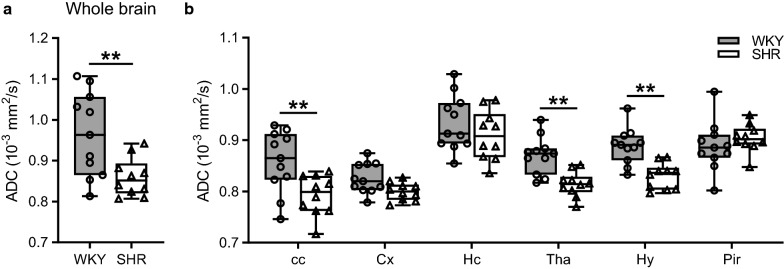
Mean ADC values of the whole brain and different anatomical structures**. a** The mean apparent diffusion coefficient (ADC) of the whole brain parenchymal tissue was significantly lower in SHR (n = 10) compared to WKY (n = 11). **b** ADC values were significantly lower in the cc, Tha and Hy of SHR. All other structures were not different between the two strains. The boxplots indicate the median and values of the 25th and 75th percentile of the data. **p ≤ 0.01, ***p ≤ 0.001 (unpaired Student’s t-test for whole brain and two-way ANOVA with Bonferroni’s post hoc tests for all other brain regions). cc, corpus callosum; Cx, cerebral cortex; Hc, hippocampus; Tha, thalamus; Hy, hypothalamus; Pir, piriform cortex

To further study the impact of hypertension on brain fluid management, we performed diffusion-weighted imaging. Whole brain apparent diffusion coefficient (ADC) values were mapped using manually drawn segmentation masks. In these masks, CSF-containing voxels were excluded since this could substantially affect the brain parenchymal ADC. We found that whole brain ADC values were significantly lower in hypertensive rats (0.86 ± 0.02 × 10^−3^ mm^2^/s) as compared to normotensive controls (0.96 ± 0.03 × 10^−3^ mm^2^/s) (Fig. 4a). Furthermore, we quantified ADC values in six different anatomical structures to assess whether there are region specific differences between the two strains. As shown in Fig. 4b, ADC values of SHR were lower in the corpus callosum (cc), thalamus (Tha) and hypothalamus (Hy).

### Localisation and total expression of Aquaporin 4

**Fig. 5:**
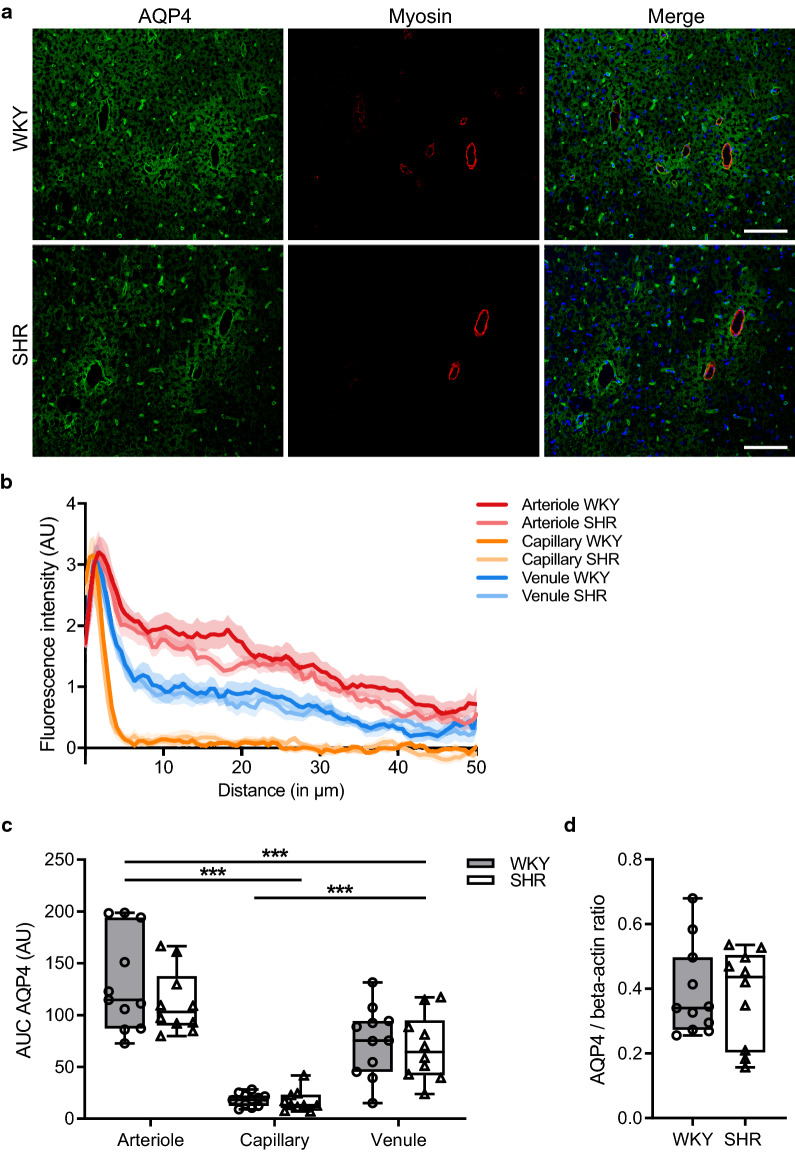
Visualisation and quantification of AQP4 expression in the cortex. **a** Representative images of AQP4 (green) and myosin (red) immunostaining in the cortex of normotensive (WKY) and spontaneously hypertensive (SHR) rats. Cell nuclei are visualized using DAPI staining (blue). **b** AQP4 fluorescence intensity profile as function of the distance from the luminal side of the vessel wall. Shown is the mean intensity profile with SEM bands for arteriole, capillary, and venule for both strains. **c** The area under the curve (AUC) of the AQP4 intensity profile of arterioles was significantly higher compared to capillaries and venules. In addition, venules showed significantly higher AUC values when compared to capillaries. AUC values of either arterioles, capillaries or venules did not differ between the two groups. **d** Total AQP4 expression in the cerebral cortex was not different between WKY (n = 11) and SHR (n = 10). The boxplots indicate the median and values of the 25th and 75th percentile of the data. ***p ≤ 0.001 (two-way ANOVA with Bonferroni’s post hoc tests for the AUC and Mann–Whitney U test for the total AQP4 expression analysis). Scale bar represents 100 µm

After MR imaging, we assessed whether the total expression and localisation pattern of aquaporin 4 (AQP4) was affected in hypertension. Brain tissue sections were immunolabelled for AQP4 and myosin, to distinguish arterioles from venules. High resolution overview images of one hemisphere were acquired using confocal microscopy. Figure 5a shows representative micrographs of the AQP4 and myosin staining of WKY and SHR animals. The intensity of AQP4 as function of the distance from either arterioles, capillaries or venules was subsequently quantified in the cerebral cortex (Fig. 5b). This showed a peak at approximately 1.5 µm from the luminal side of the vessel wall of comparable height in all three different vessel types, indicating the dense expression of AQP4 at the astrocyte endfeet that surround the vessels. An intensity profile that decreased with distance was observed for both arterioles and venules. However, the decay in intensity was steeper in venules compared to arterioles, indicating a larger area AQP4 expression around arterioles. In capillaries, the AQP4 intensity profile rapidly dropped to background levels after the initial peak. The area under the curve (AUC) was subsequently calculated to determine if the AQP4 intensity profiles were different between the different vessel types and whether there were differences between WKY and SHR, as shown in Fig. 5c. AQP4 intensities were significantly higher around arteries compared to both venules and capillaries. Next to this, venules also showed significantly higher AQP4 intensities when compared to capillaries. However, no differences between animal strains for either arterioles, capillaries and venules in AQP4 intensity were observed in the cortex.

To complement the immunohistochemical analysis of AQP4 expression around blood vessels, the total AQP4 protein expression in the cerebral cortex was measured using the ProteinSimple Wes system. The results revealed no difference in parenchymal AQP4 expression in the cortex between WKY and SHR, as shown in Fig. 5d.

**Fig. 6:**
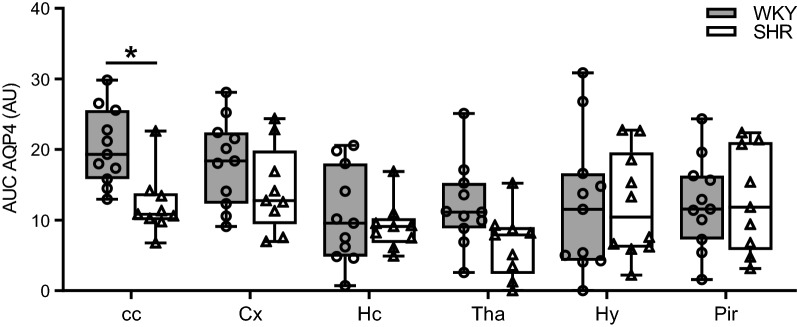
Quantification of AQP4 expression in different anatomical structures. The area under the curve (AUC) of the AQP4 intensity profile of capillaries was significantly lower in the corpus callosum in SHR (n = 10) compared to WKY (n = 11). All other structures were not different between the two strains. The boxplots indicate the median and values of the 25th and 75th percentile of the data. *p ≤ 0.05 (two-way ANOVA with Bonferroni’s post hoc tests). cc, corpus callosum; Cx, cerebral cortex; Hc, hippocampus; Tha, thalamus; Hy, hypothalamus; Pir, piriform cortex

In other anatomical brain structures, the number of arterioles and venules was smaller due to the smaller size of these structures. Moreover, in brain areas other than the cortex, most larger blood vessels are located in cisterns rather than in the parenchyma. Therefore, we only quantified AQP4 expression around capillaries in the corpus callosum, hippocampus, thalamus, hypothalamus and piriform cortex. This revealed that in the corpus callosum the AQP4 intensity was significantly lower in hypertensive rats (Fig. 6). In these same brain regions, the mean GFAP fluorescence intensity was quantified to assess reactive changes in the astroglial compartment. However, no differences in the GFAP expression were found between WKY and SHR in any of the brain structures, as shown in Additional file 2: Figure S2.

## Discussion

In this study, we investigated the effects of hypertension on changes in the water homeostasis of the brain. This was studied in spontaneously hypertensive rats and the normotensive control strain. MRI scanning revealed clear differences in both the total volume of the CSF compartment and brain parenchyma between the two groups. Next to this, hypertensive animals displayed changes in diffusional properties in various brain regions and exhibited elevated brain water contents. Immunohistochemical analysis of AQP4 showed that this protein was highly expressed around blood vessels, with a more abundant expression around arterioles as compared to capillaries and venules in both strains. In the cortex, neither quantity nor localisation of AQP4 was different between WKY and SHR. However, in the corpus callosum we observed a lower AQP4 expression around capillaries of the SHR. Taken together, these data show several differences in parameters related to water homeostasis between SHR and WKY, indicating a disrupted fluid balance in the hypertensive rodent brain.

### Brain atrophy

As we anticipated that the effects of hypertension on the brain would become more evident with aging, we studied rats at 10 months of age. We confirmed that both systolic and diastolic blood pressure, as well as the heart rate, were significantly elevated in the hypertensive rats. The body mass was similar in both groups, whereas the wet brain weight was nearly 10% less in SHR. The latter finding suggests cerebral atrophy in the hypertensive animals. Indeed, manual segmentation of T2-weighted scans revealed a decrease in intracranial volume in SHR that was comparable to the reduction based on brain weight. The reduction in brain weight was accompanied by substantially enlarged lateral and third ventricles in the hypertensive animals. The brain parenchymal volume that was subsequently calculated showed a clear difference between WKY and SHR, implying cerebral atrophy in SHR. These findings were also previously reported by the studies of Kaiser et al. [11] and Koundal et al. [17] in the same hypertensive rat model, and were also described in a number of MRI studies in hypertensive patients [18–20]. Possibly, this reflects a process where hypertension leads to chronic hypoperfusion and subsequent neurodegeneration [21]. An alternative explanation for the ventricular enlargement may be a hypersecretion of CSF. However, previous work from our group showed that the CSF production rate was not different between WKY and SHR and that SHR exhibited a normal intracranial pressure [14]. This suggests that the ventricular enlargement as observed in the current study is not consequential to an increased CSF production, but is more likely the result of ventricular expansion due to cerebral atrophy. A second alternative explanation for cerebroventricular enlargement in SHR could be that it is genetically determined, as treatment with antihypertensive medication does not normalize ventricular size [22].

### Brain tissue water content and apparent diffusion coefficient

Previous work from our group showed an altered ionic composition and a tendency towards an increased water content of the brain in the hypertensive rat [23]. Yet, a subsequent study showed an intact BBB permeability with respect to small compounds in SHR [14]. Based on these observations, in the current study we further investigated the basis of these findings and hypothesized that chronic hypertension may lead to oedema formation. Two types of oedema have been described. Cytotoxic oedema is characterized by the accumulation of ions and fluid within the cells of the brain, resulting in swelling of these cells. This rearrangement of water from the extracellular to the intracellular compartment restricts its ability to move freely within the tissue, thereby leading to a decreased diffusivity. Vasogenic oedema results from disruptions at the BBB, causing extravasation of fluids across this barrier into the brain parenchyma. As opposed to cytotoxic oedema, water molecules accumulate extracellularly in vasogenic oedema, which enhances the diffusivity as water molecules can move more easily within the brain extracellular space [24]. Based on these properties, cytotoxic oedema can be differentiated from vasogenic oedema using diffusion weighted imaging (DWI). In this study, ADC mapping of the whole brain parenchyma revealed significantly lower mean ADC values in SHR when compared to WKY. In addition, we found lower ADC values in the corpus callosum, thalamus and hypothalamus of the hypertensive animals. These results appear to be consistent with a state of cytotoxic oedema in the hypertensive rat, a condition that is normally associated with the early stages of brain ischemia after vascular occlusion. Detailed studies have shown that such reduction in ADC could be caused by a process called dendritic beading, as described in the study by Budde and Frank [25]. In response to ischemic or osmotic conditions, axons and dendrites of the central nervous system may alter their shape, transforming into bead-like structures. This process may subsequently lead to a restricted intracellular water mobility along the neurite, thereby lowering the ADC value. Yet, DWI is not sensitive enough to distinguish the changes in different cellular compartments. Accordingly, the diffusivity as measured using DWI represents a combination of both the extracellular and intracellular compartments. Thus, one alternative explanation for the current data could be a change in either the volume and/or composition of the extracellular compartment that may restrict water diffusivity, while cell volumes are normal. Another interesting possibility is that a reduction in diffusivity is directly related to a lower expression of AQP4, as will be discussed further below.

Previous work by our group showed an increased spreading of fluorescent tracers in the hippocampus of SHR, suggesting an enhanced ISF flow or tracer accessibility in this structure [23]. On the other hand, the study by Mortensen et al. found a decreased glymphatic flow in the same animal model [13]. The latter finding is mainly based on observations at the brain surface. Whether these possible alterations in either ISF or CSF flow in hypertension lead to changes in diffusivity is hard to determine, as the ADC data presented in this study reflect steady state values of diffusivity. At present, insufficient data are available to unravel this relationship and therefore additional work is needed to obtain more insight in the association between ISF flow, CSF flow, and diffusivity data.

Cerebral oedema formation was further studied by specifically measuring brain parenchymal water content by taking care not to include CSF. After desiccation of the brain tissue for one week, we found an increased percentage water content of nearly 1% in SHR compared to WKY. Even though this difference in percentage water content seems relatively small, this number can be misleading and actually reflects a larger change in brain tissue water of about 5% when normalized to dry matter. This change in water content may have major implications in terms of ionic homeostasis and the downstream neuronal function. It remains to be established whether the increased water content reflects cytotoxic oedema and how this arises in the hypertensive brain. The study by Li et al. [21] showed a reduction in blood flow and hypercapnic responses in SHR. Subsequent disturbances in the oxygen supply, and thus energy supply, might affect the function of the Na^+^/K^+^ pump [24]. As a consequence, changes in the ionic composition may develop. Previous work from our group indeed reported a shift in the whole brain Na^+^/K^+^ ratio in SHR [23]. This disruption in the ionic homeostasis may therefore be a driver for local, temporary cytotoxic oedema formation, due to cell swelling caused by the influx of Na^+^ from the extracellular space to the intracellular compartment [26, 27]. In the study by Ritz et al. [28], the gene expression profile was determined in the cortex of SHR and WKY at 9 months of age. These findings revealed that the majority of the differently expressed genes are associated with oxidative stress and ischemic responses, which indeed suggests chronic hypoxic conditions in the brains of SHR. Other studies have shown that especially the white matter is more susceptible to these ischemic conditions, as these structures are often supplied by less penetrating arterioles and capillaries [29, 30]. Since the corpus callosum, thalamus, and hypothalamus are entirely or partially composed of white matter, these brain regions may therefore be more vulnerable to hypoxia-induced oedema formation and may therefore explain the changes found in diffusivity as observed in the hypertensive rats.

### Aquaporin 4

Water homeostasis of the brain critically depends on water channels, in particular AQP4. This water channel is mainly expressed at the astroglial end-feet present at the border zones between the brain tissue and fluid compartments in the brain [31], and is thus essential for the exchange of water molecules between the parenchyma and blood. Previous studies showed that overexpression of AQP4 increases water influx into the brain when challenged, thereby promoting cytotoxic brain swelling, while deletion of this water channel reduces brain oedema formation [31, 32]. In this study, we examined the localisation pattern of AQP4 in a number of anatomical brain structures. In the cortex, we found a markedly increased expression of AQP4 in the tissue surrounding arterioles when compared to capillaries and venules. This finding was also previously reported in the study of Kress et al. [33] in the cerebral cortex of mice. It suggests that particularly arterioles have a large area of influence with respect to water exchange. However, neither Wes nor immunohistochemical analysis revealed differences in the expression level of AQP4 between SHR and WKY. This was also observed in the study of Mortensen et al. [13] in which the AQP4 distribution was quantified from immunostaining in the cortex of young and adult SHR and WKY. However, the observation that the AQP4 expression around capillaries was lower in the corpus callosum of SHR may indicate a relation between AQP4 levels and ADC values. Both AQP4 and ADC values did not differ between WKY and SHR in the cortex, while a lower AQP4 expression in the corpus callosum of SHR was paralleled by a lower ADC value when compared to its normotensive control strain. Such a correlation has been described previously in the study by Badaut et al. [34]. These authors demonstrated that after acute knock down of AQP4 expression ADC levels substantially decreased, suggesting an important role for aquaporins in facilitating water diffusion.

## Conclusions

In summary, this preclinical MRI study showed evident differences in both the total brain parenchyma volume and cerebroventricular volume between hypertensive and normotensive animals. SHR exhibited changes in diffusional properties and an increased brain parenchymal water content. This was accompanied by a reduction in AQP4 expression in the corpus callosum. These findings reveal a disrupted fluid balance in the hypertensive rat brain and may therefore provide new perspectives for future research to unravel the underlying mechanisms of high blood pressure on cerebrovascular pathology.

## Supplementary information

**Additional file 1: Figure S1.** Detection of AQP4 and beta-actin using Wes in brain lysates**. a** Representative traditional Western blot-like image of brain lysates of a WKY and SHR rat. An AQP4 signal is observed at the expected size of 33 kDa both in normotensive and hypertensive animals. The 46 kDa bands represent the beta-actin signal, which was used as a loading control. **b** Representative electropherogram of a normotensive and hypertensive rat. The peaks observed at the 33 kDa and 46 kDa correspond to the AQP4 and beta-actin signal respectively.

**Additional file 2: Figure S2.** Quantification of GFAP expression in different anatomical structures**. a, b** Representative images of GFAP (red) immunostaining in the cortex of normotensive and spontaneously hypertensive rats respectively. Cell nuclei are visualized using DAPI staining (blue). **c** The mean GFAP fluorescence intensity was not different between WKY (n=5) and SHR (n=5) in any of the anatomical brain structures (two-way ANOVA with Bonferroni’s post hoc tests). The boxplots indicate the median and values of the 25th and 75th percentile of the data. cc, corpus callosum; Cx, cerebral cortex; Hc, hippocampus; Tha, thalamus, Hy, hypothalamus; Pir, piriform cortex. Scale bar represents 50 μm.

## Data Availability

All data generated or analysed during this study are included in this published article.

## Abbreviations

Aβ: Amyloid-β
AD: Alzheimer’s disease
ADC: Apparent diffusion coefficient
AQP4: Aquaporin 4
AUC: Area under the curve
BBB: Blood-brain barrier
BP: Brain parenchyma
cc: Corpus callosum
CSF: Cerebrospinal fluid
Cx: Cerebral cortex
DWI: Diffusion weighted imaging
GFAP: Glial fibrillary acidic protein
Hc: Hippocampus
Hy: Hypothalamus
ISF: Interstitial fluid
LV: Lateral ventricle
MRI: Magnetic resonance imaging
Pir: Piriform cortex
ROI: Region of interest
SEM: Standard error of the mean
SHR: Spontaneously hypertensive rat
Tha: Thalamus
T2w: T2-weighted
WKY: Wistar Kyoto rat
3V: Third ventricle
