# Supplementary figures and images for: Altered brain fluid management in a rat model of arterial hypertension
Source: Fluids Barriers CNS. 2020 Jun 26;17:41. doi: 10.1186/s12987-020-00203-6 (PMC7318739; doi:10.1186/s12987-020-00203-6)

**A**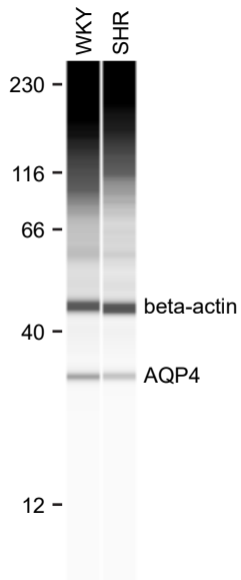**B**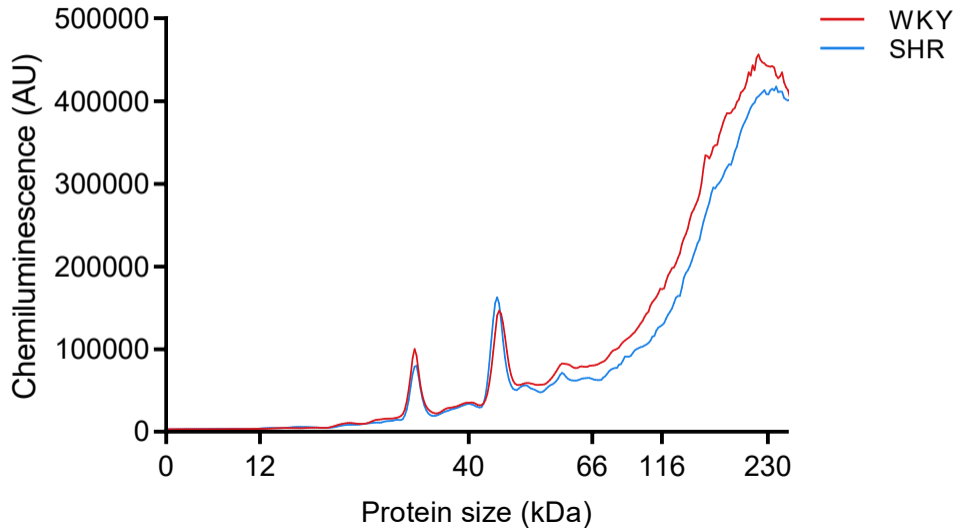

Supplement: Supplementary file 1 — Additional file 1: Figure S1. Detection of AQP4 and beta-actin using Wes in brain lysates. a Representative traditional Western blot-like image of brain lysates of a WKY and SHR rat. An AQP4 signal is observed at the expected size of 33 kDa both in normotensive and hypertensive animals. The 46 kDa bands represent the beta-actin signal, which was used as a loading control. b Representative electropherogram of a normotensive and hypertensive rat. The peaks observed at the 33 kDa and 46 kDa correspond to the AQP4 and beta-actin signal respectively. [file 12987_2020_203_MOESM1_ESM.pdf]

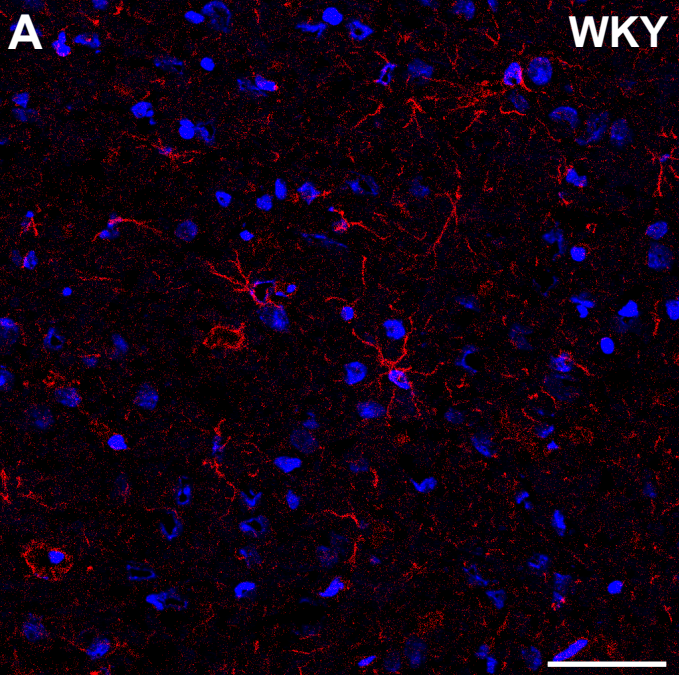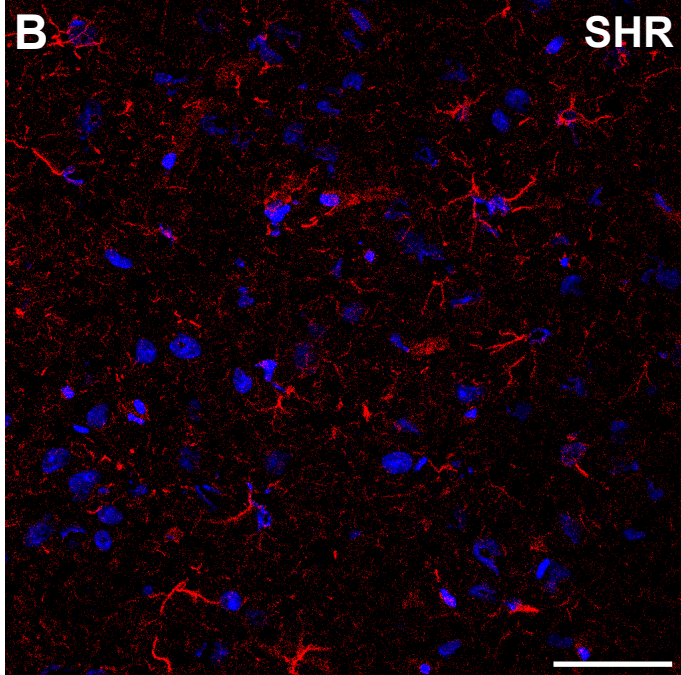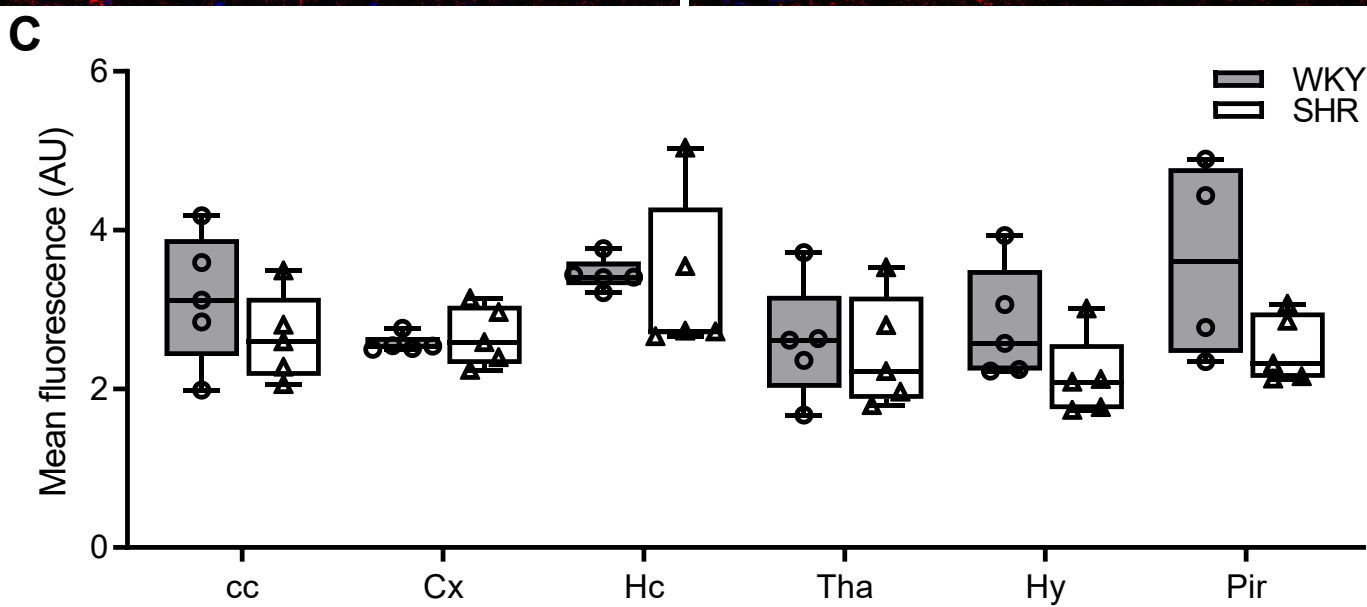

Supplement: Supplementary file 2 — Additional file 2: Figure S2. Quantification of GFAP expression in different anatomical structures. a, b Representative images of GFAP (red) immunostaining in the cortex of normotensive and spontaneously hypertensive rats respectively. Cell nuclei are visualized using DAPI staining (blue). c The mean GFAP fluorescence intensity was not different between WKY (n=5) and SHR (n=5) in any of the anatomical brain structures (two-way ANOVA with Bonferroni’s post hoc tests). The boxplots indicate the median and values of the 25th and 75th percentile of the data. cc, corpus callosum; Cx, cerebral cortex; Hc, hippocampus; Tha, thalamus, Hy, hypothalamus; Pir, piriform cortex. Scale bar represents 50 μm. [file 12987_2020_203_MOESM2_ESM.pdf]
